# Dementia risk prediction modelling in low- and middle-income countries: current state of evidence

**DOI:** 10.3389/fepid.2024.1397754

**Published:** 2024-09-18

**Authors:** Maha Alshahrani, Serena Sabatini, Devi Mohan, Jacob Brain, Eduwin Pakpahan, Eugene Y. H. Tang, Louise Robinson, Mario Siervo, Aliya Naheed, Blossom Christa Maree Stephan

**Affiliations:** ^1^ Dementia Centre of Excellence, Curtin EnAble Institute, Faculty of Health Sciences, Curtin University, Perth, WA, Australia; ^2^ Department of Psychology, King Khalid University, Abha, Saudi Arabia; ^3^ Saudi Arabian Cultural Mission in Australia, Canberra, ACT, Australia; ^4^ School of Psychology, University of Surrey, Guildford, United Kingdom; ^5^ School of Public Health, Faculty of Medicine, The University of Queensland, Herston, QLD, Australia; ^6^ Institute of Mental Health, Mental Health and Clinical Neurosciences, School of Medicine, University of Nottingham, Nottingham, United Kingdom; ^7^ Freemasons Foundation Centre for Men’s Health, Discipline of Medicine, School of Psychology, The University of Adelaide, Adelaide, SA, Australia; ^8^ Applied Statistics Research Group, Department of Mathematics, Physics and Electrical Engineering, Northumbria University, Newcastle upon Tyne, United Kingdom; ^9^ Population Health Sciences Institute, Newcastle University, Newcastle upon Tyne, United Kingdom; ^10^ Institute of Health and Society, Newcastle University Institute of Ageing, Newcastle University, Newcastle upon Tyne, United Kingdom; ^11^ School of Life Sciences, Division of Physiology, Pharmacology and Neuroscience, University of Nottingham, Queen’s Medical Centre, Nottingham, United Kingdom; ^12^ Non Communicable Diseases, Nutrition Research Division, icddr,b, Dhaka, Bangladesh

**Keywords:** dementia, risk prediction, risk prediction algorithm, low-and middle-income countries, ageing

## Abstract

Dementia is a leading cause of death and disability with over 60% of cases residing in low- and middle-income countries (LMICs). Therefore, new strategies to mitigate risk are urgently needed. However, despite the high burden of disease associated with dementia in LMICs, research into dementia risk profiling and risk prediction modelling is limited. Further, dementia risk prediction models developed in high income countries generally do not transport well to LMICs suggesting that context-specific models are instead needed. New prediction models have been developed, in China and Mexico only, with varying predictive accuracy. However, none has been externally validated or incorporated variables that may be important for predicting dementia risk in LMIC settings such as socio-economic status, literacy, healthcare access, nutrition, stress, pollutants, and occupational hazards. Since there is not yet any curative treatment for dementia, developing a context-specific dementia prediction model is urgently needed for planning early interventions for vulnerable groups, particularly for resource constrained LMIC settings.

## 1 Introduction

Dementia is a major public health priority. Globally, there are 57 million cases with most residing in low- and middle-income countries (LMICs) where resources, research, services, and support are often very limited (1). Whilst dementia is currently incurable, findings from the Lancet Commission on Dementia Prevention, Intervention and Care estimate that as many as 40% of dementia cases worldwide could be prevented by targeting 12 modifiable risk factors across the life course including early life education, midlife hypertension, obesity, hearing loss, traumatic brain injury, alcohol misuse (>21 units per week), later-life diabetes, depression, smoking, physical inactivity, social isolation, and air pollution (2). In LMICs, the potential for dementia risk reduction is even higher (3). Therefore, key research priorities are to identify modifiable risk factors, create risk scores and use this information to design interventions to delay or prevent the onset of dementia that are culturally appropriate and fit within a given healthcare context.

Over the last two decades, research into the development of prediction models to forecast dementia risk has gained momentum (4, 5). Early identification of high-risk groups is a key step in managing and potentially mitigating the impact of dementia on individuals, their families, and communities. These models include factors previously associated with dementia such as demographic (age, sex, education), health (cardio-metabolic status), social (isolation, marital status), cognitive (global and domain specific function), genetic (apolipoprotein e4 status) and lifestyle (poor diet, smoking, alcohol use, physical inactivity) variables. They have varying accuracy [i.e., with c-statistic values ranging from poor (0.49) to excellent (>0.90)] and have mixed external validity such that they do not always transport well outside the setting in which they were developed (4, 5). Further, model development and testing has almost exclusively been undertaken in high-income Western countries with predominantly White samples. Relative to high-income countries, to date, few studies have characterised dementia risk in LMICs and research into dementia prediction, prevention, and risk reduction in LMICs is limited. This is of concern given that dementia numbers are forecasted to nearly triple by 2050, with notable increases in LMIC regions of north Africa and the Middle East, and eastern sub-Saharan Africa (6).

Therefore, the aim of this mini review is to synthesise current evidence on dementia risk prediction research in LMICs. We also suggest possible next steps that could be implemented for informing the development of new strategies for dementia risk reduction and prevention in LMICs.

## 2 Methods

The results presented here are based on a synthesis of findings from three systematic reviews on dementia risk prediction model development and testing undertaken by our team. These capture all literature from database inception to 10 June 2022. Full details of each review have been published previously (see 4, 5, 7). In brief, for each of the three published reviews, Embase (via Ovid), Medline (via Ovid), Scopus, and Web of Science were searched using a combination of the following terms: dementia, Alzheimer disease, predict, develop, incident, sensitivity, specificity, ROC, area under the curve, and concordance statistic (c-statistic). Results from the electronic searches were transferred to Endnote and de-duplicated. To capture articles missed by the electronic searches, manual searches of the reference lists of all included studies was also undertaken. Titles, abstracts, and full texts were screened independently by two or more authors. Discrepancies were resolved through discussion till consensus was reached.

In all three reviews, articles were included if the study was population-based (including electronic health record data) and reported a predictive model for late-life (i.e., ≥60 years) incident dementia as the outcome, with measures of sensitivity, specificity, or discrimination (i.e., area under the curve: AUC or c-statistic). Cross-sectional studies, review articles and studies focused on clinical samples were excluded. Studies focused on young onset dementia (i.e., dementia below the age of 60 years) were also excluded. For this study, we only selected those articles that had undertaken dementia risk model development and/or testing in LMICs as defined by World Bank Criteria.

## 3 Results

### 3.1 The current state of risk model development in LMCIs

Based on a synthesis of the systematic review literature on dementia risk prediction modelling (4, 5, 7), we found that out of over 100 different models developed, only five come from studies using LMIC data. This includes studies from China [*n* = 3 studies (8–10)] and Mexico [*n* = 2 studies (11, 12)]. These models incorporate risk factors previously associated with dementia, predominantly derived from research from high-income countries (see Table 1). As shown in Table 1, model predictive accuracy ranges 0.70 [95% CI 0.64–0.73], diabetes high depressive symptoms and impairment in instrumental activities of daily living (IADL), for predicting 11-year incident dementia, in Mexico to high (c-statistic >90, for models incorporating odor identification data either alone or in combination with demographic, health, genetic and/or cognitive variables for predicting dementia over a mean follow-up of 4.9 years, in China). To date, models developed in LMICs have not been externally validated. This having important implications for cross-study comparability and raising *questions about the generalisability of the findings*. However, this is most likely due to limited availability of comparable datasets across different LMIC settings. Internal validation results, calculated using techniques such as splitting the sample into development and validation datasets, were generally good (see Table 1). These findings are consistent with those from dementia risk prediction modelling research in high-income countries.

**Table 1:** Dementia risk prediction models developed in Low- and Middle-Income Countries (LMICs).

Reference	Country	Study & whether sample is population representative [Yes/No]	*N*	Age (years)	Outcome (*n*)	Follow-up	Model variables	AUC/c-statistic (95% CI) development	AUC/c-statistic (95% CI) internal validation
Wang 2017 (9)	China	Beijing longitudinal study of aging (No)	2,788	≥60	All-cause, AD and VaD using DSM-IIIR, NINCDS-ADRDA and NINDS-AIREN criteria (*n* = 351)	7-years	Frailty index based on *n* = 40 health deficits^a^	All-cause 0.74 (0.69–0.78)	None
								AD 0.77 (0.74–0.79)	
Ding 2020 (8)	China	The Shanghai aging study (2010/11-2014/16) [No]	947	≥60	All-cause using DSM-IV criteria (*n* = 75)	Mean = 4.9-years	**M1** Age, sex, education, BMI, height, smoking, drinking, CAD, hypertension, diabetes, depression, stroke, APOE e4 &MMSE	**M1** 0.90 (0.86–0.93)	K-fold cross validation method
							**M2** Orange odour	**M2** 0.90 (0.87–0.93)	
							**M3** Leather odour	**M3** 0.91 (0.87–0.94)	
							**M4** Cinnamon odour	**M4** 0.90 (0.87–0.94)	
							**M5** Peppermint odour	**M5** 0.90 (0.87–0.94)	
							**M6** Banana odour	**M6** 0.91 (0.87–0.94)	
							**M7** Lemon odour	**M7** 0.90 (0.87–0.93)	
							**M8** Liquorice odour	**M8** 0.90 (0.87–0.93)	
							**M9** Coffee odour	**M9** 0.90 (0.87–0.93)	
							**M10** Cloves odour	**M10** 0.90 (0.87–0.93)	
							**M11** Pineapple odour	**M11** 0.90 (0.87–0.93)	
							**M12** Rose odour	**M12** 0.90 (0.87–0.94)	
							**M13** Fish odour	**M13** 0.90 (0.86–0.93)	
							**M14** OI	**M14** 0.90 (0.87–0.94)	
							**M15** Model 1 + 12 odours^b^	**M15** 0.92 (0.88–0.95)	
							**M16** Age, weight, education, depression, stroke, APOE e4, leather, peppermint, banana, lemon, pineapple, rose & MMSE	**M16** 0.91 (0.88–0.94)	
							**M17** Age, education, stroke, peppermint & MMSE	**M17** 0.90 (0.86–0.93)	
Ding 2020 (10)^c^	China	The Shanghai aging study (2010/11-2014/16) [No]	947	≥60	All-cause using DSM-IV criteria (*n* = 75)	Mean = 4.9-years	**M1** Age only	**M1** 0.84 (0.79–0.88)	**M1** 0.77 (0.65–0.89)
							**M2** Age, education, APOE e4, peppermint odour, banana odour, pineapple odour & MMSE	**M2** 0.92 (0.88–0.95)	**M2** 0.91 (0.88–0.94)
							**M3** M2 + dependency of incident dementia on all variables	**M3** 1.0 (1.0–1.0)	**M3** 0.56 (0.49–0.63)
							**M4** Age, MMSE & orange odour	**M4** 0.95 (0.93–0.96)	**M4** 0.83 (0.72–0.94)
							**M5** Age, MMSE & cinnamon odour	**M5** 0.95 (0.94–0.97)	**M5** 0.84 (0.73–0.95)
							**M6** Age, MMSE & peppermint odour	**M6** 0.95 (0.93–0.97)	**M6** 0.82 (0.70–0.93)
							**M7** Age, MMSE & pineapple odour	**M7** 0.95 (0.93–0.97)	**M7** 0.84 (0.73–0.94)
Downer 2016 (11)	Mexico	Mexican health and aging study (2001 & 2012) [Yes]	3,002	≥60	CCCE cut-off + impairment in one or more ADL or two or more IADL (*n* = 251)	11-years	**M1** Age, sex, education, hypertension, diabetes stroke, high depressive symptoms, IADL impairment, ADL impairment, fall (last 2-years) & fair-poor vision	**M1** 0.74 (0.70–0.77)	Bootstrapping
							**M2** Age, diabetes, high depressive symptoms & IADL impairment	**M2** 0.70 (0.64–0.73)	
Acosta 2018 (12)	Mexico	10/66 Study [No]	1,355	≥65	All-cause using 10/66 and DSM-IV criteria (*n* = 129)	3-years	**M1** Age**,** live in rural area, MCI, diabetes, illiteracy & 2 or more than 2 NPSs	**M1** 0.75	None
							**M2** M1 (excluding MCI)	**M2** 0.74	
							**M3** M1 (excluding NPSs)	**M3** 0.72	

AD, Alzheimer's disease; ADL, activities of daily living; APOE, apolipoprotein; BMI, body mass index; CAD, coronary artery disease; CCCE, cross-cultural cognitive examination; CLHLS, Chinese longitudinal healthy longevity survey; CVD, cardiovascular disease; DSM-IIIR, diagnostic and statistical manual of mental disorders, third edition, revised; DSM-IV, diagnostic and statistical manual of mental disorders, fourth edition; IADL, instrumental activities of daily living; M, model (e.g., M1 = Model 1); MCI, mild cognitive impairment; MMSE, mini mental state examination; NINCDS-ADRDA, National Institute of Neurological and Communicative Disorders and Stroke and the Alzheimer's Disease and Related Disorders Association; NINDS-AIREN, National Institute of Neurological Disorders and Stroke and Association Internationale pour la Recherche et l'Enseignement en Neurosciences; NPSs, neuropsychiatric symptoms (including hallucinations, delusions, anxiety aberrant motor behaviour and depression); OI, olfactory identification; VaD, vascular dementia.

^a^ The 40 deficits include arrhythmia; cataract; glaucoma; accidental urine incontinence; occurrence of falls; broken or fractured bones; tremors of hands, head, or lips; respiratory disease; arthritis; cholecystitis; neurasthenia; gastric diseases; cervical syndrome; constipation; recurrent headache; vertigo; recurrent headache; chest pain after activity; kidney disease; sudden loss of consciousness; sudden loss of speech; sudden paralysis or weakness; osteoporosis; chronic diarrhea; absence of clear hearing when talked to; dental problems; using a walking stick; help with eating; help with grooming; help with dressing; help with getting on/off bed; help with bathing; help with moving around in the house; help with cooking meals; help with managing your money; help with taking a bus; help with shopping; help with walking a 300-mdistance; help with walking up and down stairs; and, depression [Centre for Epidemiological Studies Depression (CES-D) measure].

^b^ Olfactory identification test includes orange, leather, cinnamon, peppermint, banana, lemon, liquorice, coffee, cloves, pineapple, rose, and fish.

^c^ Key findings are reported here. For the full set of risk modelling results please see the original publication.

Dementia risk model development in LMICs has used the same methodology (e.g., Logistic and Cox Regression Modelling) and generally the same set of predictor variables as used in research from high-income countries. However, given contextual differences it is likely that different methods will be needed to accurately predict dementia risk in these settings. Indeed, as highlighted in Table 1, research into model development in LMICs has not considered inclusion of other key risk factors that are likely to be important determinants of dementia in these settings including, for example, socio-economic variables (e.g., income and life-long socio-economic disadvantage), poor infant health (e.g., low-birth weight), healthcare access (including access to treatment and medications e.g., for cardio-metabolic diseases), nutritional profiles (including food security and dietary patterns), stress, pollutants (e.g., air, water and noise), and occupational hazards (e.g., pesticide exposure). Further, to be scalable risk models must include data that can be captured in a cost-effective way utilising available infrastructure. Understanding cross-cultural differences in risk factors and risk levels is critical for developing strategies for dementia prevention and care across different socio-economic contexts (13).

### 3.2 External validation of dementia risk models developed in high-income countries in LMIC settings

When models that have been developed in high-income countries are tested in LMICs including sites in China, Cuba, Dominican Republic, Mexico, Peru, Puerto Rico, and Venezuela external validation results are mixed (11, 14). Five models have been tested including the following:

1. Brief Dementia Screening Indicator [BDSI] incorporating age, education, stroke, diabetes, underweight (body mass index (BMI) < 18.5), needing assistance with managing finances or taking medications and depressive symptoms (developed using USA data).
2. Study on Aging, Cognition and Dementia model [AgeCoDe] incorporating age, subjective memory impairment, verbal fluency, delayed recall, MMSE, and instrumental activities of daily living (developed using data from Germany).
3. Cardiovascular Risk Factors, Aging, and Incidence of Dementia (CAIDE) Dementia Risk Score CAIDE risk score incorporating age, sex, education, systolic blood pressure, BMI, total cholesterol, and physical activity (developed using data from Finland).
4. Basic Dementia Risk Model [BDRM] incorporating age, stroke, subjective memory decline and needing help with finances or medication (developed using data from the Netherlands).
5. The Australian National University Alzheimer's Disease Risk Index [ANU-ADRI] incorporating age group (by sex), education, BMI, diabetes, symptoms of depression, total cholesterol, traumatic brain injury, smoking, alcohol use, social engagement, physical activity, cognitive activity, fish intake, and pesticide exposure (developed using an evidence synthesis approach).

**Figure 1:**
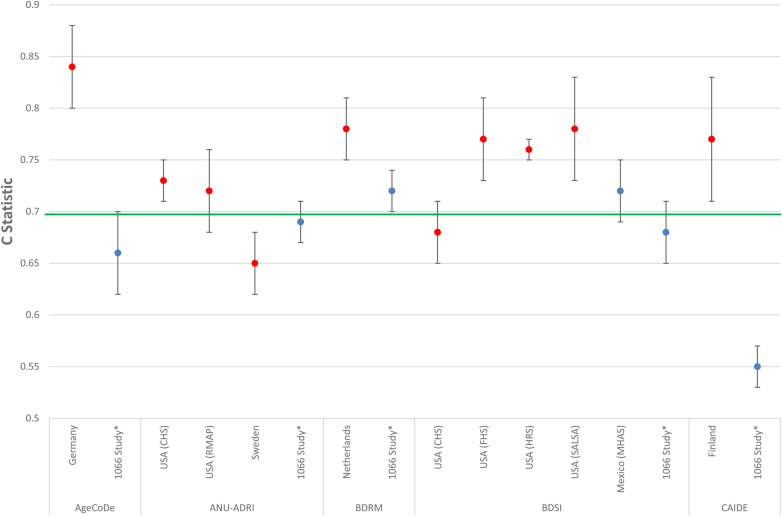
Comparison of the predictive accuracy of models developed in high income countries when mapped in low- and middle-income countries (LMICs). Blue dot = LMIC results. Red dot = High income country results. Green line = Minimum c-statistic cut-off (i.e., 0.70) for a model to be considered clinically meaningful. * Results are from a meta-analysis incorporating data from the 10/66 Study from seven low- and middle-income countries (LMICs) including China Cuba, Dominican Republic, Mexico, Peru, Puerto Rico, and Venezuela. 10/66, 10/66 study; AgeCoDe, Study on Aging, Cognition and Dementia; ANU-ADRI, Australian National University Alzheimer's Disease Risk Index; BDRM, Rotterdam Study Basic Dementia Risk Model; BDSI, Brief Dementia Screening Indicator; CAIDE, Cardiovascular Risk Factors, Aging and Dementia; CHS, Cardiovascular Health Cognition Study; FHS, Framingham Heart Study; HRS, Health and Retirement Survey; MHAS, Mexican Health and Aging Study; RMAP, Rush Memory and Aging Project; SALSA, Sacramento Area Latino Study on Aging; USA, United States of America. Figure made for this manuscript utilising results from Stephan et al. (14).

Only two of these models, the BDSI and BDRM, had reasonable accuracy when tested in LMICs as shown in Figure 1. Poor transportability of models developed in high-income settings to LMICs may be due to several reasons. First, the meaning of risk factors such as low education and the strength of association between a risk factor and incident dementia is likely to vary across settings. Therefore, models may need to be recalibrated by re-weighting a given predictor depending on location. Second, methodological differences, for example, in sample selection, follow-up time, dementia diagnostic criteria and risk factor assessment may have also influenced the results. Third, the models may be missing key indicators of risk unique to LMIC settings as highlighted above (e.g., socio-economic disadvantage, food insecurity, low birth weight, healthcare accessibility etc.). Last, is the issue of bias (e.g., sampling, resource and contextual) when applying models developed in HICs in LMIC settings that could impact model predictive accuracy in addition to feasibility, acceptability and appropriateness. More in-depth analysis to explore variation in risk (e.g., demographic, lifestyle, heath, genetic/ethnic diversity, healthcare access etc.) and the most relevant risk factors across different socioeconomic and cultural contexts will ensure that models are appropriately tailored. This should take a bi-directional approach whereby researchers in both high-income and LMIC settings collaborate closely, sharing unique insights and accounting for the full spectrum of risk factors across different contexts to enhance health equality.

### 3.3 Next steps

Lowering risk and preventing dementia in LMICs is an urgent priority. Achieving this will require government and community support and action driven by context-specific evidence. Based on the findings to date we suggest the following next steps:

1. LMICs need to invest into the collection of data to build the evidence base on dementia and its risk factors and accelerate research into the development of context-relevant risk prediction and risk reduction programs. Indeed, while dementia research is growing in LMICs it still lags high-income countries in terms of investment, availability of data and published literature.
2. New models, incorporating LMIC specific risk variables, need to be developed that are resource driven e.g., include the least number of predictor variables that are affordable and can be easily obtained in the setting that the model is to be applied.
3. Any further dementia risk prediction and reduction research should be undertaken in consultation with consumers, academics, healthcare providers, policy makers and other key stakeholders, from the setting in which the risk model is to be applied, to ensure that it is culturally sensitive, sustainable, and addresses the specific preferences, needs and challenges of the target population.

Building the evidence based on the unique profile of risk and protective factors for dementia in LMICs will be important for developing culturally appropriate intervention strategies to mitigate the burden of disease associated with dementia in low resource settings worldwide.

## 4 Conclusion

Further work is needed into dementia risk prediction model development to underpin the design and testing of dementia risk reduction and prevention trials in LMICs. As highlighted in this review, research into dementia risk prediction is expanding in LMICs paving the way for new opportunities to advance understanding about dementia and its risk factors in these contexts. While China and Mexico are at the forefront of this area of research, there is a substantial gap in representation across other LMICs. Strategic expansion to other LMICs would facilitate a more comprehensive understanding of the determinants influencing dementia risk in varying socio-cultural, economic, and environmental settings. Such knowledge is especially important within the context of rapid demographic transitions, including ageing populations and increased dementia risk in LMIC settings to inform the development of new strategies to reduce risk, delay onset, and prevent dementia.
